# The evaluation of *Suchana*, a large-scale development program to prevent chronic undernutrition in north-eastern Bangladesh

**DOI:** 10.1186/s12889-020-08769-4

**Published:** 2020-05-22

**Authors:** Nuzhat Choudhury, Mohammad Jyoti Raihan, S. M. Tanvir Ahmed, Kazi Eliza Islam, Vanessa Self, Shahed Rahman, Lilly Schofield, Andrew Hall, Tahmeed Ahmed

**Affiliations:** 1 Nutrition and Clinical Services Division, icddrb, 68 Shaheed Tajuddin Ahmed Sarani, Mohakhali, Dhaka, 1212 Bangladesh; 2 Department of Anthropology, Durham University, Durham, UK; 3 Save the Children Bangladesh, House CWN (A) 35, Road 43, Gulshan, Dhaka, Bangladesh; 4 Save the Children U.K., 1 St John’s Lane, London, EC1M 4AR UK

**Keywords:** Randomized controlled trial, Stepped wedge design, Nutritional program evaluation, Malnutrition

## Abstract

Evidence of the impact of community-based nutrition programs is uncommon for two main reasons: the lack of untreated controls, and implementation does not account for the evaluation design. *Suchana* is a large-scale program to prevent malnutrition in children in Sylhet division, Bangladesh by improving the livelihoods and nutrition knowledge of poor and very poor households. *Suchana* is being implemented in 157 unions, the smallest administrative unit of government, in two districts of Sylhet. *Suchana* will deliver a package of interventions to poor people in about 40 randomly selected new unions annually over 4 years, until all are covered. All beneficiaries will receive the normal government nutrition services. For evaluation purposes the last 40 unions will act as a control for the first 40 intervention unions. The remaining unions will receive the program but will not take part in the evaluation. A baseline survey was conducted in both intervention and control unions; it will be repeated after 3 years to estimate the impact on the prevalence of stunted children and other indicators. This stepped wedge design has several advantages for both the implementation and evaluation of services, as well as some disadvantages. The units of delivery are randomized, which controls for other influences on outcomes; the program supports government service delivery systems, so it is replicable and scalable; and the program can be improved over time as lessons are learned. The main disadvantages are the difficulty of estimating the impact of each component of the program, and the geographical distribution of unions, which increases program delivery costs. Stepped implementation allows a cluster randomized trial to be achieved within a large-scale poverty alleviation program and phased-in and scaled-up over a period of time. This paper may encourage evaluators to consider how to estimate attributable impact by using stepped implementation, which allows the counterfactual group eventually to be treated.

## Background

In 2015, the World Health Assembly set a target to reduce by 40% the number of stunted children worldwide by 2025 [1]. The prevalence of childhood stunting in Bangladesh is coming down by about 1.5% per year [2] and the country is almost on target, but the prevalence shows substantial variation around the country. In the last Bangladesh Demographic and Health Survey, 49.6% of children less than 5 years of age in Sylhet division were stunted compared with 28.1% in Khulna division, a difference of over 20 percentage points [2]. Because of concern for this disparity, Save the Children (SC) designed and sought funding in partnership with other agencies for *Suchana*, a 7-year program that aims to prevent chronic malnutrition by delivering a package of nutrition-specific interventions through the government system plus nutrition-sensitive development interventions delivered by international and local non-government organizations (NGOs) to up to 250,000 poor and very poor households in two districts of Sylhet division.

The aim of the *Suchana* program, beyond improving the nutrition and growth of children in the target households, is for successful elements to be replicated, scaled up and sustained while informing policies and other programs. The program has itself been built on lessons learned from other large projects such as Stimulating Household Improvements Resulting in Economic Empowerment (SHIREE) [3] and the first Most Critical Days Program (MCDP) in Bangladesh. The design was also informed by Household Economy Analysis, Cost of the Diet assessments [4], formative research, political economy analysis and social protection mapping. To ensure the program is replicated robust evidence is required [5]. This requires a counterfactual, so the program needs to be designed in a way that allows a concurrent control group to be achieved in the same geographic area at the same time who have similar characteristics. This challenge is one of the main reasons for the lack of robust evidence of the impact of large scale programs on children’s nutritional status [6]. There is also a need to collect data on indicators of nutrition that minimise recall errors and social desirability bias so as to prevent over reporting of good behaviour [7].

This paper describes the design of the evaluation of *Suchana*, focusing on how the requirement for a counterfactual was balanced with the needs of a humanitarian development program, and discusses the advantages and disadvantages of the design.

## Design

**Fig. 1:**
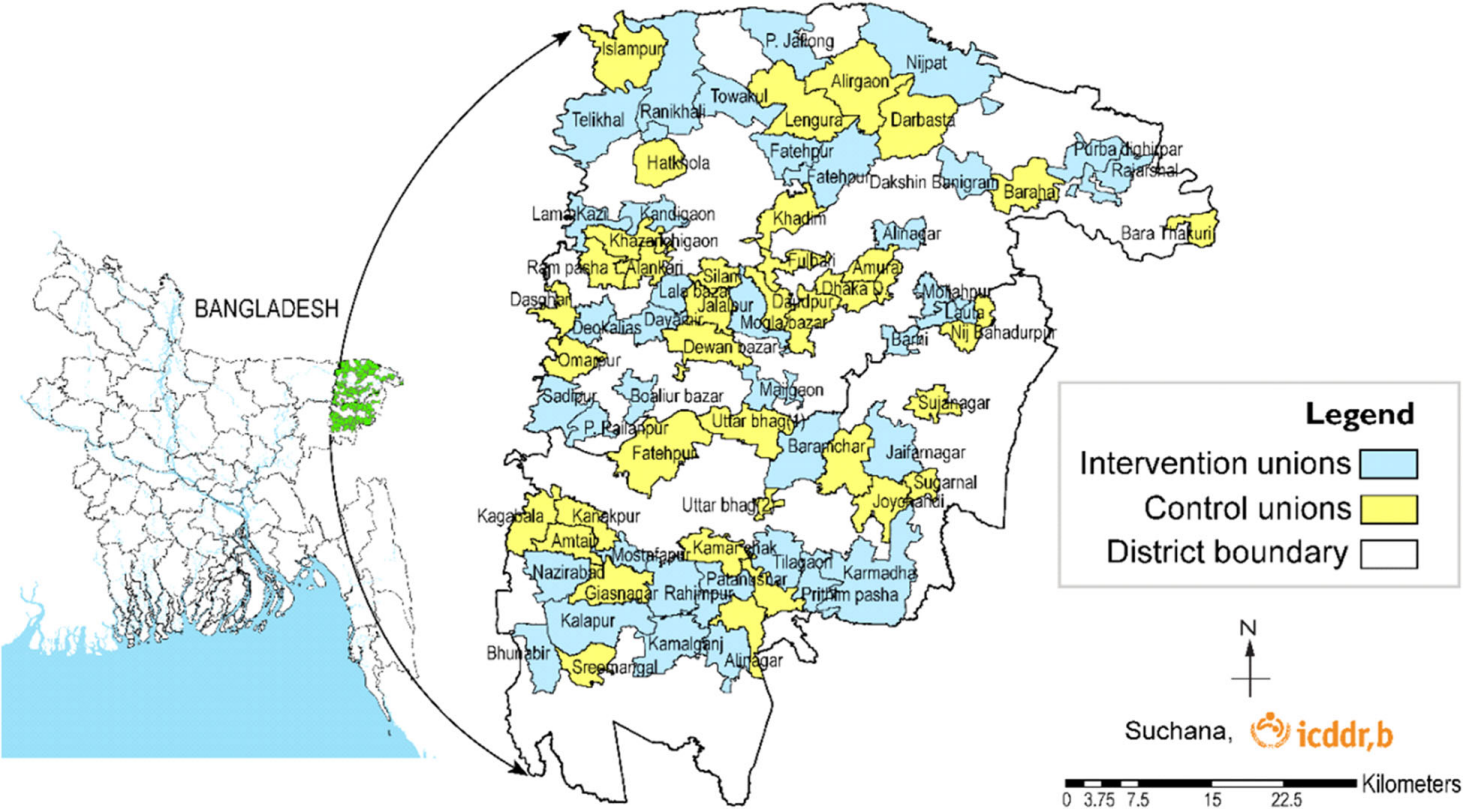
A map of the two districts in Sylhet division showing the unions randomly selected for phase 1 of *Suchana* (intervention) and phase 4 (control). The figure was created by the authors

*Suchana* is a large-scale development program being implemented in Sylhet division of north-eastern Bangladesh by a consortium of agencies, including technical, implementing and research partners: SC, WorldFish, Helen Keller International, International Development Enterprises, Rangpur Dinajpur Rural Service Bangladesh, Friends in Village Development Bangladesh, Center for Natural Resource Studies, the icddr,b (formerly known as the International Centre for Diarrhoeal Disease Research, Bangladesh) and led by SC (Fig. 1). *Suchana* aims to enrol up to 250,000 poor and very poor households in the program with the primary intention of preventing chronic undernutrition and linear growth retardation among children under 2 years of age born into these households during the program.

### Suchana program elements

There are five main elements to the program: (i) improved nutrition governance that translates political commitments into practice; (ii) an effective and inclusive government service delivery system that enhances access to, and demand for, nutrition-related services to include the promotion of government nutrition services for pregnant women, mothers, and young children; (iii) economic empowerment of the very poor, particularly women and adolescent girls, so that they can overcome economic barriers to better nutrition; (iv) improving the knowledge, skills and power of women and adolescent girls to enable them to challenge harmful cultural practices and gender norms including gender-based violence, early marriage and early pregnancy that can affect mother and child nutrition; and (v) robust evidence of the impact of the package of interventions to prevent chronic malnutrition which will inform the policies and practices of the government and other agents of change.

### Suchana program implementation and evaluation

To be able to estimate the attributable impact of the package of interventions provided by *Suchana* and to control for the effect of other programs and factors in the same geographical area at the same time, a stepped wedge design for implementation was proposed by SC. This design is particularly suited to evaluations of services, typically through government units of delivery such as the sub-district [8]. In this instance it is the union, the lowest tier of government in Bangladesh. This design allows the intervention to be phased-in and scaled-up over a period of time so that the beneficiaries in the last group of unions can act as an untreated control group until they join the program in the last phase, while the beneficiaries in the first phase of unions receive the interventions over a period of time sufficient to lead to a difference in outcome measures. This period is 3 years for the beneficiaries of *Suchana*. Using a pre post design for evaluation, a baseline survey before the program begins will serve to check that the outcome measures are not significantly different in children in potential target households in the intervention and control unions, and the survey will be repeated after 3 years intervention, at the same time of year to control for seasonality and economic fluctuations, to assess whether the null hypothesis, of no difference between children in the intervention and control unions, can be rejected. The evaluation will be done only at the household level since the major outcome is to reduce the prevalence of stunted children. The *Suchana* program itself has a separate monitoring, evaluation, accountability and learning (MEAL) system to assess changes in other program elements in the community and at the national level.

The validity of any conclusions requires that the units of delivery are randomly allocated to the groups or phases. The phased or ‘stepped’ process of implementation enables a cluster randomized controlled trial to be achieved without having a permanently untreated control group, as the last phase of unions will eventually join the program for 3 years. The approach is also preferable from an ethical point of view as every person involved in the research also benefits from the program. All households in all unions are eligible to receive nutrition-specific interventions provided through the Government’s National Nutrition Service (NNS) throughout the program, but these are promoted strongly by the program in the intervention unions. The design allows other effects of *Suchana* to be assessed providing that there is a plausible causal pathway between the interventions and a sensitive and specific outcome variable that is expected to change, and providing that the sample size is sufficient.

The design does not allow the impact of each individual component of *Suchana* to be estimated, but it may be possible to assess if the degree of participation in different elements of the program is associated with an outcome, by an analysis of variance of indicators pertaining to participation. The indicators of exposure will include the monetary value of investments in livelihoods such as fish or poultry farming, the income generated by these and other livelihood activities, and participation in behaviour change activities, such as the number of sessions attended by the mother of a child. Data was collected on household conditions, assets and circumstances, as they may also modify the outcome of *Suchana*. The collected data focused on: household characteristics and assets; access to land and water bodies; water, sanitation and environmental hygiene; household dietary diversity and food security; income expenditure, savings and loans; coping strategies, participation in other programs and income generating activities; health; access to government facilities and services; women’s reproductive history, knowledge and empowerment; men’s knowledge on child feeding and pregnancy care; and the characteristics of adolescents; engagement with market actors and access to social protection.

### Union assignment

**Fig. 2:**
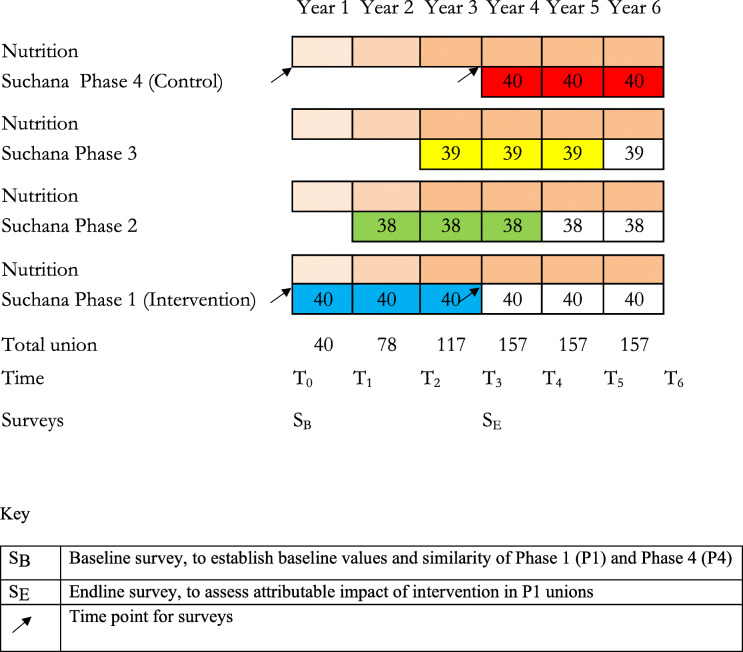
The *Suchana* evaluation diagram

The basis of assessing the impact is that the only difference between intervention and control households is, on average, due to the interventions delivered and supported by *Suchana*. Two districts of Sylhet division, Sylhet and Maulvibazar, were selected for the program. They were chosen because no other large-scale food security and nutrition or mother and child nutrition programs were being implemented and they were not substantially within the ‘hoar’ wetlands, where the climatic and geographic conditions would make implementation difficult at this large scale and within a fixed timeframe. All 157 unions were within these two districts, excluding commercial tea gardens and a small number of urban areas. The unions were randomly allocated into four phases in a lottery, which took place in the presence of program staff, government staff and local elected representatives of unions and dignitaries. This level of transparency was critical for community and government acceptance and all the union representatives understood that all unions would receive the program at some point. Forty unions were randomly allocated to phase 1 and 40 unions to phase 4, to achieve equal numbers for comparison; the other 77 unions were randomly allocated to phases 2 and 3. Each phase of *Suchana* will last 3 years (36 months) so that, by the end of the third year, all but the last phase will have joined the program and the households in phase 1 will have benefited from 3 years of interventions. This process provided the intervention and control unions for the evaluation. This design is shown in Fig. 2. As it is expected that all unions will be similar and the clusters were randomized to avoid any bias, Phase 2 and 3 were not considered in the evaluation.

### Beneficiary selection

Villages in each union were selected for the program based on their vulnerability to poverty and natural disasters such as lack of development programs, remoteness and risk of flooding. This was done by a process of discussions with local government officials, representatives of unions, and experts, and field visits by *Suchana* staff. In each village field staff used a participatory approach including focus group discussions and individual interviews with local people to identify the poor and very poor households to be the beneficiaries in each community. The wealth ranking sessions usually took place in the courtyard of a prominent household, which is a convenient location to congregate approximately 150–200 households in each village. Using a process of participatory rural appraisal, households were allocated to one of four community-defined wealth groups. If any villages contained more than 200 households, two wealth ranking sessions were conducted, each joined by half of the household heads. The households ranked in the two poorest wealth groups were physically verified then visited by program field staff to validate that they met the inclusion criteria to be selected for *Suchana*. A household was enrolled in the program, if any one of the following five conditions was satisfied: i) the household members, throughout the year were unable to eat three meals a day or ii) the total monthly income was less than BDT. 7500 [USD 1 =~ BDT 85] or iii) the total business assets of the household was worth no more than BDT. 15,000 (excluding homestead land and pond or dyke) or iv) the homestead land of the household was no more than 10 decimals or v) cultivable land was no more than 50 decimals. Additionally, all households satisfied one of the following four conditions: i) has a married women between 15 to 45 years-of-age or ii) has a pregnant women, including divorcees and widows or iii) has at least one child under 2 years-of-age or iv) has at least one female living in the household and aged between 15 to 19 years [9].

It is to be noted that since the ability to implement new or scaled-up livelihoods and homestead gardens by a household would likely depend on their degree of poverty, the program provide assets to the poorest to ensure that all beneficiaries would be in a position to engage in livelihood activities and take equal advantage of the participatory training offered. It is expected that the ratio of asset-transfer to non-asset transfer beneficiaries will be 1:1.5.

### Sample size for evaluation

The primary outcome indicator is the mean z-score of height-for-age from which the percentage of children who are stunted (z-score < − 2) is derived. The secondary indicators are mostly related to infant feeding: how long after delivery the child was first breastfed; whether the child was exclusively breastfed for the first 6 months of life; whether a child aged > 1 year is still being breastfed; the age at which solid, semisolid, or soft foods were introduced; the diversity of the diet in the last 24 h; the number of meals in the last 24 h; whether a minimum acceptable diet was given; whether iron-rich or iron-fortified foods were given [10]; and the haemoglobin concentration. Although, *Suchana* is working with households which contain a married women aged between 15 to 45 years or the household has a pregnant women or the household has at least one female adolescent, for the purposes of evaluation only households with at least one child under 2 years-of-age was selected, as the primary aim of the evaluation was to assess changes in the prevalence of stunted children among *Suchana* household beneficiaries.

A calculation was done to estimate the sample size of children required, in three age groups: 0–5 months, 6–11 months and 12–23 months. This age range was chosen to be able to assess the impact of interventions in the first 1000 days after conception, the period during which undernutrition typically has its greatest effect on linear growth.

For children aged 0–5 months the calculation was based on the prevalence of exclusive breastfeeding (EBF) of 55.3% from an unpublished study in Sylhet district, an intra-cluster correlation coefficient (ICC) of 0.0293, an assumed increase of EBF of 10% points, a power of 80% and a level of significance of 0.05. The sample size estimated was 520 subjects per group so the minimum total sample size for the baseline survey was 1040 and for the final survey was 2080, to allow for disaggregation of data into groups which did and did not receive asset transfers.

For children aged 6–11 months the primary outcome indicator was optimal infant and young child feeding (IYCF) practices. Using the information from the same unpublished survey which estimated a prevalence of 12.6%, an ICC of 0.0235, an assumed increase of 7%, and the same power and level of significance the sample size was estimated at 600 per arm, so 1200 in total and 2400 in the final survey, to allow for disaggregation.

For children aged 12–23 months the sample size calculation was based on the main derived outcome indicator, the percentage of stunted children. In order to detect a difference of 6% points between the intervention and the untreated control group in 40 unions in each group after 3 years, the sample size required was estimated to be 1520 children in each group assuming a power of 80%, 5% precision, 40 clusters per group and an ICC of 0.01. Due to refusals or data collection errors, the sample size was estimated 3200 in total for baseline survey, and 6400 in the final survey to allow for disaggregation.

Simple randomization was used to allocate the unions to four different phases or clusters. A baseline survey was carried out among beneficiaries of *Suchana* in 40 Phase 1 unions (intervention) and among potential beneficiaries in 40 phase 4 unions (control) in 2017. The survey included children aged 0 to 23 months and their parents, plus 1200 adolescent boys and 1200 girls aged 15 to 19 years to assess their knowledge of health and nutrition, to assess their decision making power, and to estimate the proportion who are already married, though the sample of the adolescents were themselves unmarried. Systematic sampling was used to select beneficiary or potential beneficiary households to be assessed using the *Suchana* beneficiary/potential beneficiary list as the sampling frame. The final survey will be carried out in 2020. The evaluation was registered at the Registry for International Development Impact Evaluations (RIDIE-STUDY-ID-5d5678361809b) on16/08/2019, before starting the end-line survey.

### Other large scale programs and evaluation design

Programs adopt an evaluation design which best fits the program context and objectives, and the funding available. Some designs aim for plausible results rather than requiring statistical probability [11]. PROCOMIDA was a health and nutrition program to prevent stunting in Guatemala [12]. The evaluation of the program was unique in having both cross-sectional and a longitudinal cluster randomized components, with the primary aim to assess changes in IYCF practices and the nutritional status of beneficiary children [11, 12]. Another large scale nutrition program, called Alive & Thrive [5] also used a mixed design. In two countries, Bangladesh and Vietnam, cluster randomized probability designs were used while in Ethiopia an adequacy design was applied. A key challenge was the rapid scale-up of operations and the difficulty of identifying a valid comparison group in each situation [5]. The stepped wedge approach to implementation offers a potential solution which enables the random allocation of groups of beneficiaries [13]. Stepped implementation favours large programs within a public health system [14]. This design can help to mitigate some usual threats during implementation, such as staff turnover, although it cannot be completely avoided or controlled [15]. A notable intervention delivered in a clustered and randomized stepped manner was the Gambian hepatitis intervention study [16]. In this study, which began in 1986, a vaccination program was rolled out in different phases in randomly allocated geographic areas. Although the program took 4 years to complete national coverage the follow-up of the each cohort was an ongoing process [16]. A stepped wedge design was used in 2003 to evaluate the Mexican universal health insurance program in which 74 matched pairs of clusters were chosen for the intervention and to serve as a control [17]. The design protected the evaluation from unexpected or untimely political interventions. Health facilities can serve as the unit of randomisation. For example in the Netherlands a trial of digression management was undertaken in 17 nursing homes [14].

### Suchana stepped wedged design

The stepped wedge design to implement *Suchana* allowed a randomized cluster-controlled trial of a package of interventions that has been designed to improve livelihoods, provide income, inform and empower girls and women, and improve the nutritional status of their children in area of Bangladesh that is lagging behind the country in an important indicator of national development, the percentage of stunted children.

A program cannot easily be delivered to beneficiaries in individual households and at the same time have similar neighbours as controls, as this could lead to contamination or resentment. It also does not test the government service delivery mechanism. The best option is to deliver the program through the smallest unit of government, which in Bangladesh is the union. The staff of the union can then deliver the interventions to all beneficiaries in their union, selected on the basis of need by the community themselves using participatory methods, and the population understands that all beneficiaries in that union have been chosen based on those criteria. The program and the evaluation use the smallest unit of service delivery by the government, so the processes developed can be replicated and scaled up, while the union also provides rational clusters for the evaluation. The design is consistent with normal government processes.

### Counterfactual

The design allows for a counterfactual while delivering a program in a phased approach that is eventually received by all communities. Governments are rarely able to implement a new program in all areas from the same start date, so a phased approach allows the program to be scaled up over time. The rationale for this is understood by program staff, which is why it needs to be integrated into the design from the start of the planning process.

This method of program delivery allows a counterfactual, which is provided by a control group of future beneficiaries in 40 unions. This allows changes that would have occurred anyway to be subtracted from the change in the beneficiaries in the intervention group, and thus estimate the impact that is attributable to the intervention. The prevalence of stunted children is generally going down in Bangladesh, but with the program it should to go down more. The measurement of a single group before and after the program, the typical method used to evaluate program [18], would not allow this subtraction, so a counterfactual is needed. A similar program in Bangladesh called SHOUHARDO, which combined livelihoods with education, empowerment and nutrition-specific interventions, claimed to have caused a rapid reduction in the prevalence of stunting of 15.7 percentage points over a period of 3 years [19] but crucially had no counterfactual. The evaluation of SHOUHARDO was based on data from surveys before and after the program, and studied small numbers of children selected in clusters.

### Ethical issues

There is no untreated control group in this design and no placebo is needed (or possible). Every union gets the program eventually, so there are no ethical issues due to withholding treatment. Randomization is crucial, as in all controlled trials, to distribute differences between the units of delivery in the intervention and control groups. As the smallest unit of delivery of services by the government is the union, this was identified as the unit of delivery of *Suchana* and the unit of randomization for the evaluation, so creating a cluster randomized controlled trial. The randomization process, by which the phases of involvement of unions in the program was decided, was done transparently, with representatives of the unions present as observers, so they could see that there is no bias in the selection process, which is sometimes the case in programs.

### Design allows learning

All unions will receive the program for the same number of years but, as the program is delivered in annual phases, but it can be changed for unions in phases 2, 3 and 4 as lessons are learned and improvements are made. It is not a fixed intervention, unlike a normal randomized controlled trial of a standard treatment, even if the interventions delivered to households in phase 1 are sustained. This is good for the program, which can be developed based on learning and experience, while the evaluation of its impact on the first beneficiaries continues.

The rate of reduction in the prevalence of stunted children is quite slow, even without a program; to achieve a difference between children in intervention and control unions that is statistically significant takes time. Three years was estimated to be the minimum period needed to show a difference and this influenced the scale of the program, the number of unions necessary for a cluster randomized trial, and the total budget, both for the program and the evaluation.

### Close link between evaluators and program managers

The advantages and benefits of the proposed evaluation design led the donors to fund a separate evaluation both in terms of the evaluators, to ensure objective and independent data were collected, and to fund it separately, so that it did not come out of the funding for the main program. This separation enhances credibility and strengthens the process. There was also close consultation between program managers and evaluators during the planning of implementation and during service delivery. The challenges of implementing a large and complex program led to delays which had to be accommodated by the evaluation agency, so flexibility was required to undertake the baseline survey. The evaluation instruments were grounded in the specific program theory of each component of the intervention, to capture and record access to *Suchana* interventions, as well as to measure and capture contextual factors among *Suchana* workers, mothers, households, and communities, all of which could influence the effectiveness of the interventions.

### Disadvantages

There are however several noted disadvantages of a stepped wedge design in the context of program implementation [14, 15]. In the case of *Suchana*, the randomization process led to unions in phase 1 that are distributed throughout two districts, which increases the travel time of project staff from their base office to program unions, increases the cost of training government staff if they have to travel outside their unions to a central training venue, and increases program support costs. This makes it hard to estimate the cost effectiveness of implementing the program, as it will be higher than it need have been, and it also increases evaluation costs for the same reasons. The most cost-efficient way to implement a program would be to involve unions in contiguous blocks close to the project office in the first phase, and then spread out across the two districts, also in blocks. The issue was partially addressed by stratifying unions by geographical location before randomization.

The randomization process increases the risks of contamination of people in control unions from people in intervention unions, especially with public health messages that are specific to the program. Due to the scale of the program in terms of the number of unions required to achieve a sufficient number for a stepped wedge design and for a controlled evaluation, the program is expensive. However, considering the costs per beneficiary, the potential impact on households, the impact on the government delivery systems, and the potential evidence generated, the program arguably provides better value-for-money than a traditional delivery approach, which cannot yield results of the same magnitude or potential value. The number of clusters in an evaluation is equally as important as the sample size of subjects studied, in order to control for inter-cluster differences. The *Suchana* program will cost GBP 50 million over 7 years while the evaluation will cost around GBP 1.5 million. This is a substantial investment by donors, but it could generate high quality evidence. It is not an appropriate or possible design for all evaluations of programs to improve government services.

The design does not easily allow the separate impact of each element of a package of interventions to be estimated. Development programs rarely deliver a single, pure intervention, such as a drug, and typically contain several elements, some supporting each other, while others may be independent of each other but may still be nutrition-sensitive. The only way to assess whether an element contributes to the difference in outcomes is to try to measure indicators of participation in the program or the quantity of intervention received, if it can be measured, and factors that might influence uptake and utilization by households. The challenge for the evaluators is to identify the indicators with the implementers and then make sure that they are recorded accurately throughout the program. This requires planning and coordination.

It is also a challenge to identify potential beneficiaries in control unions during the baseline survey who have the same characteristics as potential beneficiaries in the intervention unions, and then not provide interventions. The same criteria used by the implementers to identify beneficiaries need to be used by evaluators to identify subjects for study in all unions in both surveys. At the final survey this is easier as the subjects for study could be included in the program when it begins in phase 4.

The main element of the program that is not evaluated by the stepped wedge design is the process of implementation. This is being conducted through the MEAL system of *Suchana*, which has a separate evaluation design and evaluation process, so that all elements of the process related to the evaluation are separated from the current evaluation design.

The randomization of 157 unions in two Districts of northern Bangladesh into four phases has allowed an evaluation to be designed to estimate the impact of interventions intended to prevent chronic malnutrition in children during the first 1000 days of children’s lives. The timing of the evaluation is also important because, unlike in most programs in which the impact evaluation is completed at the end of the program and by the time the results are available the program has finished, *Suchana* will be able to actively use its impact evaluation for program learning and adjustment but also for advocacy to government and other partners for 3 years before the program finishes. The baseline evaluation started during November 2016 and ended in February 2017. This evaluation design is not suitable or feasible for all programs but, when it is finished in 2020, it may constitute one of the largest ever randomized controlled cluster trials of efforts to prevent stunting ever attempted.

## Conclusions

A stepped wedge design for implementation was used to *Suchana* which is particularly suited to evaluations of service delivery. The design allows the intervention to be phased-in and scaled-up over a period of time. The phased or ‘stepped’ process of implementation enables a cluster randomized controlled trial to be achieved without having a permanently untreated control group. However, the design does not allow the impact of each individual component of intervention to be estimated. But the design of the evaluation of *Suchana* and the lessons learned in doing so, which will be reported, could help and encourage other evaluators to consider this design in the right circumstances, and improve the quality and value of program evaluations.

## Data Availability

Data sharing is not applicable to this article as no datasets were generated or analysed for the current study.

## Abbreviations

EBF: Exclusive breastfeeding
ICC: Intra-cluster correlation coefficient
IYCF: Infant and young child feeding
SC: Save the Children
